# Supplementary material for: End Sequence Analysis Toolkit (ESAT) expands the extractable information from single-cell RNA-seq data
Source: Genome Res. 2016 Oct;26(10):1397–410. doi: 10.1101/gr.207902.116 (PMC5052061; doi:10.1101/gr.207902.116)
Supplement: Supplemental Material [file supp_gr.207902.116_Supp_Materials.tar.gz › ESAT-esat_v0.1/doc/html/classbroad_1_1core_1_1sequence_1_1_fasta_parser.html]

ESAT: broad.core.sequence.FastaParser Class Reference


ESAT


- Main Page
- Related Pages
- Packages
- Classes
- Files

- Class List
- Class Index
- Class Hierarchy
- Class Members

All Classes Namespaces Files Functions Variables Enumerator Pages

- broad
- core
- sequence
- FastaParser

Public Member Functions |
List of all members

broad.core.sequence.FastaParser Class Reference

Inheritance diagram for broad.core.sequence.FastaParser:

[legend]

Collaboration diagram for broad.core.sequence.FastaParser:

[legend]

|  |  |
| --- | --- |
| Public Member Functions | |
| void | parse (InputStream in, FastaHandler handler) throws IOException |
|  | |
| Public Member Functions inherited from broad.core.sequence.AbstractFastaParser | |
| String | getCurrentSequenceId () |
|  | |
| long | getOffset () throws IOException |
|  | |
| long | getFileSize () |
|  | |
| char | getCurrentBase () |
|  | |

|  |  |
| --- | --- |
| Additional Inherited Members | |
| Static Public Attributes inherited from broad.core.sequence.AbstractFastaParser | |
| static final int | BUFFER\_SIZE = 8192 |
|  | |
| Protected Member Functions inherited from broad.core.sequence.AbstractFastaParser | |
| void | setCurrentSequenceId (String currentSequenceId) |
|  | |
| Protected Attributes inherited from broad.core.sequence.AbstractFastaParser | |
| long | fileSize |
|  | |
| long | offset |
|  | |
| char | currentBase |
|  | |

## Member Function Documentation

|  |  |  |  |
| --- | --- | --- | --- |
| void broad.core.sequence.FastaParser.parse | ( | InputStream | *in*, |
|  |  | FastaHandler | *handler* |
|  | ) |  | throws IOException |

Here is the call graph for this function:

---

The documentation for this class was generated from the following file:

- java/broad/core/sequence/FastaParser.java


---

Generated on Fri Aug 15 2014 15:10:55 for ESAT by  

 1.8.7
